# 

**Published:** 1875-07

**Authors:** 


### CONTENTS:

ORIGINAL COMMUNICATIONS:

ART. I. Cerebral Meningitis (Intercurrent.) By Walter Hay, M.D., Chicago ..... 481

ART. II. Case in Practice. By B. F. Farley, M.D., Chebanse, Ill. ..... 485

ART. III. Intestinal Hæmorrhage in an Infant. By Norman Bridge, M.D., Chicago ..... 487

ART. IV. Femoral Hernia. By J. McLean, M.D., DuQuoin, Ill. ..... 488

ART. V. Cancer of Pylorus, etc. By Drs. J. H. Etheridge, and I. N. Danforth, Chicago ..... 489

ART. VI. Cases. By R. L. Rea, M.D., Chicago ..... 495

ART. VII. Foreign Body in Orbit. By M. G. Sloan, M.D., Delmar, Ia. ..... 497

PROGRESS IN MEDICAL SCIENCES:

ART. I. Progress of Obstetrics. By E. Warren Sawyer, M.D., Chicago ..... 500

REPORTS OF SOCIETIES ..... 503

SELECTIONS ..... 525

EDITORAL ..... 552

MORTALITY STATISTICS, Chicago ..... 559

